# Supplementary figures and images for: Characterisation and chemometric evaluation of 17 elements in ten seaweed species from Greenland
Source: PLoS One. 2021 Feb 8;16(2):e0243672. doi: 10.1371/journal.pone.0243672 (PMC7869976; doi:10.1371/journal.pone.0243672)

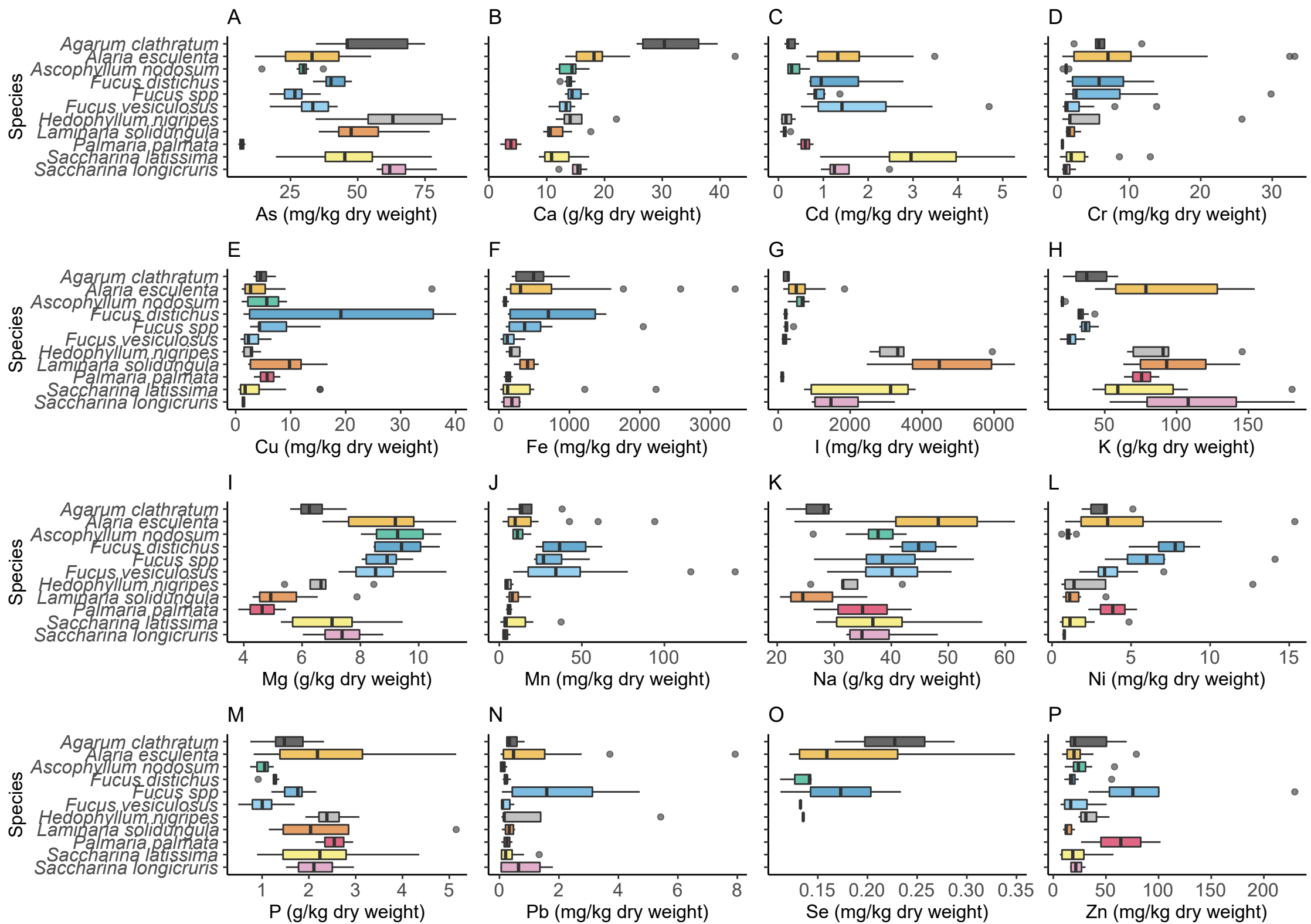

Supplement: S2 File — (PDF) [file pone.0243672.s002.pdf]
