# Supplementary material for: Characterisation and chemometric evaluation of 17 elements in ten seaweed species from Greenland
Source: PLoS One. 2021 Feb 8;16(2):e0243672. doi: 10.1371/journal.pone.0243672 (PMC7869976; doi:10.1371/journal.pone.0243672)
Supplement: Attachment — Submitted filename: Response_to_reviewers.pdf [file pone.0243672.s004.pdf]

Yi Hu
PLOS ONE

20 January 2021
KJK

Response to reviewers

We would like to thank the reviewers for taking the time to review our manuscript carefully. We have taken great care to respond to each of the comments and believe the manuscript has been improved. Our answers to each of the raised points are indicated by >.

Academic editor

1.) PLOS ONE's style requirements

> We have followed PLOS ONE's style requirements exactly, using the LaTeX template provided by PLOS ONE and checking all figures through PACE.

2.) Details regarding participant consent

> Businesses were contacted through their official email addresses available from their homepages, and we collected no personal data. The identity of the businesses and the results were anonymised. We have clarified this in the methods section.

3.) Figure 1 – possible copyright of map images

> Katharina J. Kreissig created the illustration in Inkscape. The map outline used is modified from the public domain figure "Arctic-umiaq-line-ports-of-call.svg" - downloaded from Wikimedia commons here:

https://commons.wikimedia.org/wiki/File:Arctic-umiaq-line-ports-of-call.svg (Last accessed 2021-01-14). We have included a separate statement about the figure with our resubmission.

Reviewer #1:

Keywords are missing?

> We would like to refer to page 1 of the compiled first submission "PONE-S-20-45496.pdf", where keywords are listed.

Introduction

Lines 32-36. Please, add the name of the authors to the scientific names of the species the first time you cite them.

REG-no. DK 30 06 09 46

DTU Food
National Food Institute
Kemitorvet
Building 204
2800 Kgs. Lyngby
Denmark
Tel. +45 35 88 70 00
kjkr@food.dtu.dk
www.food.dtu.dk

> We have incorporated this suggestion into our revision.

Line 44. Please, add your hypothesis at the end of the Introduction section. May be you want to extend your Introduction a bit to justify your hypotheses.

> We have incorporated this suggestion into our revision.

Materials and methods.

Lines 138-139. Please, provide the name of the restaurants and the seaweed producer in supplemental material.

> We have intentionally anonymised the results. The materials and methods section has been adjusted to reflect this. No personal information was collected, and the identity of the restaurants/producer had no impact on the result (i.e., average portion size of seaweed salad).

Results

Lines 162-166. This paragraph should go in Material and Methods.

> We have incorporated this suggestion into our revision.

Lines 167-169. This paragraph should go in Material and Methods (Statistical analyses).

> We have incorporated this suggestion into our revision.

Lines 310-311. You haven't proved this to say: 'The observed natural variation at a given sampling site is due to different factors, both abiotic (e.g. salinity) and biotic (e.g. fouling)'. You should always write in conditional tense if you have not proved what you are commenting in this sentence and although the text.

> We have incorporated this suggestion into our revision.

I think it would be clearer if Results would be shown separately from the Discussion

> We respectfully disagree. We believe that for this article, it is better to have a combined results and discussion section, as there otherwise would be extensive repetition of element concentrations. Also, we respectfully refer to the author guideline for the journal, which permits the use of a combined results and discussion section.

Tables

Tables 2 and 4 should be converted into one or more figures including different histograms, where it would be easier to see the differences between species (as you have done in Figure 4. Actually Figure 4 seems redundant with Table 2?). May be Table 4 could be converted in a stacked bar or a circle diagram.

> Table 2 is a central result of the study. Readers of the article will be interested in these exact numbers to compare with results from their own studies.

> However, to also accommodate the reviewer's great suggestion and as a service to the readers we have now included boxplots in the supplementary materials figure A to provide a graphical overview of the results presented in Table 2.

> Figure 4 shows more detailed information about different thallus parts for selected seaweed species. This information is not available in Table 2, which shows median contents of the entire macroalgae.

> Table 4 is very information dense, and we do not feel we can represent it appropriately as a figure without loss of overview of the data.

Table 4. Please indicate how many grams of seaweeds would include the salad.

> We have incorporated this suggestion into our revision.

I have not properly corrected the English writing since it is not my native language.

Reviewer #2

> We have incorporated all of your suggestions into our revision. They were very helpful. Thank you for your help.

Best regards,

Katharina Johanna Kreissig
PhD student
DTU Food
