# Supplementary material for: Characterisation and chemometric evaluation of 17 elements in ten seaweed species from Greenland
Source: PLoS One. 2021 Feb 8;16(2):e0243672. doi: 10.1371/journal.pone.0243672 (PMC7869976; doi:10.1371/journal.pone.0243672)
Supplement: Attachment — Submitted filename: PLOS ONE review comments.docx [file pone.0243672.s003.docx]

**The reviewer’s comments on Manuscript Number PONE-D-20-36449 are tabulated as follows:**

| **S/№** | **Manuscript line №** | **On the manuscript** | **Reviewer’s comment (change to)** |
| --- | --- | --- | --- |
| 1 | Before line 63 (page 3/20) | table 1. | **change to:**  Table 1. |
| 2 | Line 93 (page 3/20) | between 0 ng mL^-1^ and 400 ng mL^-1^ | **change to:** between 0 and 400 ng mL^-1^ |
| 3 | Lines 100 - 101 (page 3/20) | from 0 ng mL^-1^ to 400 ng mL^-1^ | **change to:** from 0 to 400 ng mL^-1^ |
| 4 | Lines 116 - 117 (page 5/20) | between 0 ng mL^-1^ and 100 ng mL^-1^ | **change to:** between 0 and 100 ng mL^-1^ |
| 5 | Between lines 244 and 245 (title of Fig 4. on page 8/20) | the first quartile, the median,  and the third quartile, | **change to:** the first quartile, median, and third quartile, |
| 6 | Lines 254 - 263 (page 9/20)  [* change so as to curtail repetitions and give a clearer presentation] | Pétursdóttir and colleagues [37] reported similar concentrations for total As in stipes of Icelandic *A. esculenta* (53 mg kg^-1^ ± 3 mg kg^-1^ their study, 45 mg kg^-1^ ± 6 mg kg^-1^ our study), however they reported much higher concentrations in the midrib (43 mg kg^-1^ ± 4 mg kg^-1^ their study, 23 mg kg^-1^ ± 7 mg kg^-1^ our study) and the blade (93 mg kg^-1^ ± 4 mg kg^-1^ their study, 31 mg kg^-1^ ± 16 mg kg^-1^ our study) than we found.  With regard to As content in *S. latissima*, Pétursdóttir and colleagues [37] reported somewhat lower concentrations for total As in stipes of Icelandic *S. latissima* (53 mg kg^-1^  ± 4 mg kg^-1^ their study, 75 mg kg^-1^ ± 32 mg kg^-1^ our study), however they reported much higher concentrations in the blade (old frond 117 mg kg^-1^ ± 9 mg kg^-1^ and young  frond 116 mg kg^-1^ ± 6 mg kg^-1^ their study, 53 mg kg^-1^ ± 7 mg kg^-1^ our study). | **change to:** The comparison of reports on the concentrations (mg kg^-1^) of total As in Icelandic *A. esculenta* from the Pétursdóttir et al. [37] and present studies showed for: stipes (53 ± 3 and 45 ± 6); midrib (43 ± 4 and 23 ± 7); and blade (93 ± 4 and 31 ± 16) respectively.  With regards to the As content (mg kg^-1^) in Icelandic *S. latissima*, the Pétursdóttir et al. [37] and present studies reported for: stipes (53 ± 4 and 75 ± 32); and blade ([117 ± 9 – old frond and 116 ± 6 – young frond] and 53 ± 7) respectively. |
| 7 | Line 396 (page 13/20) | diet (2 g day^-1^ to 3 g day^-1^), | **change to:**  **diet (2 to 3 g day^-1^),** |
| 8 | Line 401 (page 13/20) | see also table 3, | **change to: see also** Table 3, |
